# Enhancing Metabolic Engineering in Medicinal Plants Through Prime Editing

**DOI:** 10.1111/pbi.70532

**Published:** 2026-01-06

**Authors:** Haomiao Yu, Xiao Feng, Xiaohang Zheng, Xiao Wang, Wenxin Zheng, Zhizhou Zhang, Yuanyuan Jiang, Ruiwu Yang, Li Zhang, Zhaohui Zhong

**Affiliations:** ^1^ College of Science Sichuan Agricultural University Ya' an Sichuan China; ^2^ State Key Laboratory of Crop Gene Exploration and Utilization in Southwest China, Rice Research Institute Sichuan Agricultural University Chengdu Sichuan China; ^3^ College of Life Science Sichuan Agricultural University Ya' an Sichuan China; ^4^ Synthetic Biology and Genome Editing Center, Rice Research Institute Sichuan Agricultural University Chengdu Sichuan China

**Keywords:** medicinal plants, metabolic engineering, prime editing

Tanshinone and phenolic acid are key therapeutic compounds in the medicinal plant *Salvia miltiorrhiza* , while rutin is the major bioactive metabolite in the medicinal plant *Fagopyrum dibotrys*. However, their natural levels in cultivated varieties remain low, limiting their pharmacological potential. Enhancing metabolite accumulation through the modification of upstream transcription factors and key biosynthetic enzymes has shown promise (Deng et al. 2020). Compounding the challenge, medicinal plants like *S. miltiorrhiza* are perennials with long life cycles, making conventional breeding inefficient. While CRISPR‐Cas9 has facilitated gene knockout strategies in medicinal plants, precise base editing technologies remain underutilised (Das et al. 2024). Prime editing (PE) has emerged as a powerful tool for introducing targeted nucleotide changes; thus, offering a promising route for molecular breeding (Anzalone et al. 2019). While their application in medicinal plants remains largely unexplored.

To address these limitations in genome engineering of medicinal plants, we develop the prime editor in medicinal plants. Our previous work demonstrated that N‐terminus M‐MLV RT fused to Cas9 nickase plus epegRNA prime editor (NEPE) is highly efficient in rice (Zhong et al. 2024), indicating that the N‐terminal fusion of M‐MLV reverse transcriptase is particularly well‐suited for plant applications. Building on this, and with optimization of the expression system, we further adapted the prime editor for medicinal plants, termed MediPlant‐NEPE (Figure 1a; Figure S1).

**FIGURE 1:**
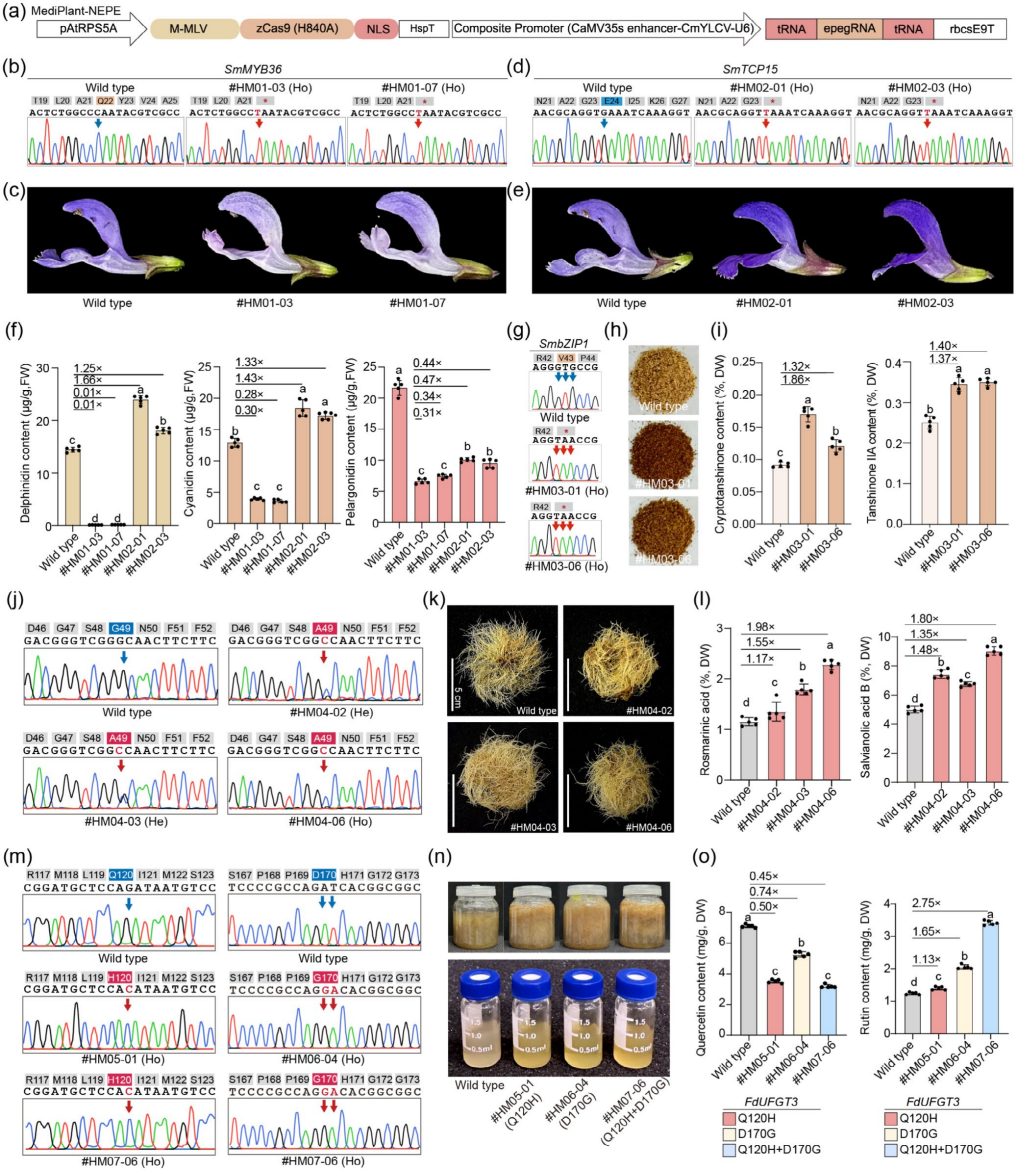
Construction and application of MediPlant‐NEPE for enhancing metabolic engineering in medicinal plants. (a) Schematic of MediPlant‐NEPE. (b) Genotyping of *SmbZIP1*‐edited hairy roots, red arrows indicate edits. (c) Phenotypes of *SmMYB36* homozygous mutants. (d) Genotyping of *SmTCP15*‐edited lines, red arrows indicate edit sites. (e) Phenotypes of *SmTCP15* homozygous mutants. (f) HPLC quantification of anthocyanin in mutant flowers. (g) Genotyping of *SmbZIP1*‐edited lines, red arrows indicate mutation sites. (h) Dry root powder of *SmbZIP1* mutants. (i) HPLC quantification of major tanshinones in roots. (j) Genotyping of *SmRAS*‐edited hairy root lines, amino acid changes shown above chromatograms. (k) SmRAS^G49A^ hairy roots after 56 days in liquid medium. (l) HPLC quantification of phenolic acids in *SmRAS* mutants. (m) Genotyping of *FdUFGT3*‐edited *F. dibotrys* hairy roots, red arrows indicate edits. (n) Hairy root cultures (top) and methanol extracts (bottom) of wild‐type and transgenic *F. dibotrys*. (o) HPLC quantification of quercetin and rutin in *FdUFGT*3 mutants.

We first targeted *SmMYB36* and *SmTCP15* in *S. miltiorrhiza* to test the efficiency of MediPlant‐NEPE and investigate their roles in flower pigmentation. Targeted C>T conversion at Q22 in *SmMYB36* and a G>T conversion at E24 in *SmTCP15* induced premature stop codons. Following Agrobacterium‐mediated transformation, positive transgenic lines were obtained with mutation frequencies of 21.21% for *SmMYB36* and 21.21% for *SmTCP15* (biallelic mutation rates of 15.15% and 12.12%, respectively; Figure 1b,d; Table S1). *SmMYB36* biallelic mutants exhibited lighter flowers, while *SmTCP15* biallelic mutants showed deeper coloration (Figure 1c,e). HPLC analysis revealed significant changes in anthocyanin content: *SmMYB36* mutants had a drastic decrease in delphinidin and a 30% reduction in cyanidin and pelargonidin, while *SmTCP15* mutants showed a 1.66‐fold increase in delphinidin and a 1.43‐fold increase in cyanidin (Figure 1f; Figure S2a,b). Unlike previous reports (Hsu et al. 2024), *SmMYB36* mutants did not exhibit albino flowers, likely due to reduced *SmANS* expression (Figure S2c). In contrast, SmTCP15 mutants had a 2.3‐ to 2.6‐fold increase in *SmANS* expression (Figure S2d). This study provides new insights into the role of *SmTCP15* as a negative regulator of anthocyanin biosynthesis and demonstrates the potential of prime editing for fine‐tuning phenotypic traits.

We next applied MediPlant‐NEPE to enhance secondary metabolite production in *S. miltiorrhiza* by targeting *SmbZIP1*, a key regulator of tanshinone and phenolic acid biosynthesis. A GTG>TAA mutation was introduced at residue V43, generating a stop codon, resulting in a mutation frequency of 26.47% and a biallelic mutation rate of 17.65% (Figure 1g). Homozygous mutants displayed deeper root colour (Figure 1h; Figure S3a) and increased tanshinone content (Figure 1i; Figure S3b). Specifically, cryptotanshinone and tanshinone IIA levels increased by 1.86‐fold and 1.40‐fold, respectively, while tanshinone I showed no significant change. The phenolic acid content was decreased in *SmbZIP1* mutants (Figure S3c,d), consistent with previous reports (Deng et al. 2020). We also targeted *SmRAS* in hairy roots to enhance rosmarinic acid and salvianolic acid B production (Figure S4a). The G49A mutation, generated by a G>C conversion, achieved an editing efficiency of 39.47% and a biallelic mutation rate of 15.79% (Figure 1j). Two heterozygous lines (#HM04‐02, #HM04‐03) and one homozygous line (#HM04‐06) were selected for further analysis (Figure 1k). HPLC confirmed that the homozygous mutant #HM04‐06 exhibited a 1.98‐fold increase in rosmarinic acid and a 1.80‐fold increase in salvianolic acid B (Figure 1l). All homozygous mutants showed a 1.93‐fold increase in rosmarinic acid and a 2.07‐fold increase in salvianolic acid B (Figure S4b–e). Molecular docking revealed that the G49A mutation minimises the enzyme pocket size and creates new interactions with the substrate and product (Figure S4f). These results demonstrate that SmRAS^G49A^ enhances both rosmarinic acid and salvianolic acid B content.

We further applied the MediPlant‐NEPE system to enhance rutin content in the wild species *F. dibotrys*, aiming to facilitate its rapid domestication as a medicinal plant. The *UFGT3* gene, a key enzyme in rutin biosynthesis (He et al. 2022) (Figure S5a) Amino acid substitutions at positions 120 (Q120H) and 170 (D170G) of the FdUFGT3 protein were predicted to enhance enzymatic activity and increase rutin production (Figure S5b). Using MediPlant‐NEPE, we introduced these mutations, achieving editing efficiencies of 12.00% for Q120H, 8.00% for D170G, and 12.00% for the double mutant with both Q120H and D170G (Figure 1m). Homozygous mutant lines were selected for further analysis (Figure 1n). HPLC analysis showed a decrease in quercetin levels across all three lines (Figure 1o; Figure S5c), with the double mutant exhibiting a 2.75‐fold increase in rutin content, while single mutations showed limited improvement. These results demonstrate that MediPlant‐NEPE can efficiently enhance secondary metabolite production through enzyme engineering. Molecular docking revealed that the mutations altered the FdUFGT3 enzyme pocket, with the single mutation causing quercetin and UDP‐glucose to bind in separate pockets (Figure S5d). In contrast, the double mutation enlarged the enzyme pocket to accommodate both substrates and the product rutin (Figure S5e). These findings highlight the potential of MediPlant‐NEPE for improving metabolite production in medicinal plants.

Overall, MediPlant‐NEPE provides a proof‐of‐concept for prime editing in the medicinal plants *S. miltiorrhiza* and *F. dibotrys*. Our results demonstrate its potential for broader application in other medicinal species and highlight its promise for molecular breeding, enhancement of secondary metabolites, and germplasm innovation, thereby accelerating the development of improved cultivars and shortening breeding cycles for economically and therapeutically valuable plants.

## Author Contributions

Z. Zhong. and L.Z. designed the experiments. H.Y., X.F. and X.Z. generated all the constructs. X.W. and W.Z. generated stable transgenic lines and analysed the plants. Z. Zhang., Y.J. and R.Y. conducted the HPLC assays. Z. Zhong., L.Z. and H.Y. wrote the paper with input from other authors. All authors read and approved the final manuscript.

## Funding

This work was supported by the National Natural Science Foundation of China, 32301245. China National Postdoctoral Program for Innovative Talents, BX20240244. Sichuan Science and Technology Program, 2024JDRC0010. National modern agricultural industry technology system Sichuan innovation team, SCCXTD‐2024‐19.

## Conflicts of Interest

The authors declare no conflicts of interest.

## Supporting information


**Appendix S1:** pbi70532‐sup‐0001‐AppendixS1.xlsx.zip.

**Appendix S2:** pbi70532‐sup‐0002‐AppendixS2.docx.

## Data Availability

The data that supports the findings of this study are available in the Supporting Information of this article.

## References

[pbi70532-bib-0001] Anzalone, A. V. , P. B. Randolph , J. R. Davis , et al. 2019. “Search‐and‐Replace Genome Editing Without Double‐Strand Breaks or Donor DNA.” Nature 576: 149–157. 31634902 10.1038/s41586-019-1711-4 PMC6907074

[pbi70532-bib-0002] Das, S. , M. Kwon , and J. Y. Kim . 2024. “Enhancement of Specialized Metabolites Using CRISPR/Cas Gene Editing Technology in Medicinal Plants.” Frontiers in Plant Science 15: 1279738. 38450402 10.3389/fpls.2024.1279738 PMC10915232

[pbi70532-bib-0003] Deng, C. , M. Shi , R. Fu , et al. 2020. “ABA‐Responsive Transcription Factor bZIP1 Is Involved in Modulating Biosynthesis of Phenolic Acids and Tanshinones in *Salvia miltiorrhiza* .” Journal of Experimental Botany 71: 5948–5962. 32589719 10.1093/jxb/eraa295

[pbi70532-bib-0004] He, M. , Y. He , K. Zhang , et al. 2022. “Comparison of Buckwheat Genomes Reveals the Genetic Basis of Metabolomic Divergence and Ecotype Differentiation.” New Phytologist 235: 1927–1943. 35701896 10.1111/nph.18306

[pbi70532-bib-0005] Hsu, C. T. , C. C. Chiu , P. Y. Hsiao , et al. 2024. “Transgene‐Free CRISPR/Cas9‐Mediated Gene Editing Through Protoplast‐To‐Plant Regeneration Enhances Active Compounds in *Salvia miltiorrhiza* .” Plant Biotechnology Journal 22: 1549–1551. 38174833 10.1111/pbi.14285 PMC11123421

[pbi70532-bib-0006] Zhong, Z. , T. Fan , Y. He , et al. 2024. “An Improved Plant Prime Editor for Efficient Generation of Multiple‐Nucleotide Variations and Structural Variations in Rice.” Plant Communications 5: 100976. 38751122 10.1016/j.xplc.2024.100976 PMC11412927

