# Supplementary material for: Enhancing Metabolic Engineering in Medicinal Plants Through Prime Editing
Source: Plant Biotechnol J. 2026 Jan 6;24(5):2858–60. doi: 10.1111/pbi.70532 (PMC13110134; doi:10.1111/pbi.70532)
Supplement: Supplementary file 2 — Appendix S2: pbi70532‐sup‐0002‐AppendixS2.docx. [file PBI-24-2858-s002.docx]

**Enhancing metabolic engineering in medicinal plants through** **prime editing**

Haomiao Yu^1, Ɨ^, Xiao Feng^1, Ɨ^, Xiaohang Zheng^2, Ɨ^, Xiao Wang ^2^, Wenxin Zheng ^1^, Zhizhou Zhang ^1^, Yuanyuan Jiang ^1^, Ruiwu Yang ^3^, Li Zhang^1 *^, Zhaohui Zhong ^2, 4 *^

^1^ College of Science, Sichuan Agricultural University, Ya’ an 625015, Sichuan, China.^2^ State Key Laboratory of Crop Gene Exploration and Utilization in Southwest China, Rice Research Institute, Sichuan Agricultural University, Chengdu 611130, Sichuan, China.^3^ College of Life Science, Sichuan Agricultural University, Ya’ an 625015, Sichuan, China.^4^ Synthetic Biology and Genome Editing Center, Rice Research Institute, Sichuan Agricultural University, Chengdu 611130, Sichuan, China.

^Ɨ^ These authors contributed equally to this work.

***Corresponding authors:**

Li Zhang, College of Science, Sichuan Agricultural University, Ya’ an 625015, Sichuan, China; Email: [zhangli@sicau.edu.cn](mailto:zhangli@sicau.edu.cn)

Zhaohui Zhong, State Key Laboratory of Crop Gene Exploration and Utilization in Southwest China, Rice Research Institute, Sichuan Agricultural University, Chengdu 611130, Sichuan, China, Email: [zhaohuizhong@sicau.edu.cn](mailto:zhaohuizhong@sicau.edu.cn)

**SUPPLEMENTARY INFORMATION**

**Supplemental Materials and Methods.**

**Figure S1. Plasmid map in this study.**

**Figure S2. Analysis of *SmMYB36* and *SmTCP15* mutant lines.**

**Figure S3. *SmbZIP1* mutants enhances metabolite production of tanshinones in *Salvia miltiorrhiza*.**

**Figure S4. SmRAS^G49A^ enhances metabolite production in *Salvia miltiorrhiza* hairy roots.**

**Figure S5. FdUFGT3 variants enhance rutin biosynthesis in *Fagopyrum dibotrys* hairy roots.**

**Figure S6. Sanger sequencing results of additional mutant lines generated in this study.**

**Figure S7. Fresh root weight of mutant lines generated in this study.**

**Supplementary Tables**

**Table S1. Prime editing efficiency of MediPlant-NEPE system.**

**Table S2. Details of prime editing sequences in this study.**

**Table S3. Oligos used in this study.**

**Supplemental Materials and Methods**

**Vector construction**

The *AtRPS5A* promoter was amplified from *Arabidopsis thaliana* Col-0 genome according to accession AP002040.1 with forward primer (5’-CTCCCCATAATCGTGAGTAGATATA-3’) and reverse primer (5’- GGCTGTGGTGAGAGAAACAGAGCGT-3’). The composite promoter was synthesis by Sangon Biotech (Shanghai, China) according to previous report. The MediPlant-NEPE backbone pLSD801 was generated by Gibson Assembly. Target site in this study was list on **Table S2.** The PBS and RTT sequences were designed according to guidelines established in previous study(Zhong et al., 2024). The epegRNA was synthesis according to the design and assembled into pLSD801 through Golden Gate reaction(Engler et al., 2009). Oligos for PCR and qPCR used in this study was list on **Table S3**.

**Agrobacterium-Mediated Transformation and Regeneration of Hairy Roots and Transgenic Plants**

The transformation assay was performed as previous described(Yu et al., 2025). Sterile leaves were pre-cultured on 1/2 MS solid medium for 3 days at 25 °C. Mechanical wounding was performed using a sterilized scalpel, followed by immersion in an *Agrobacterium tumefaciens* C58C1 suspension (OD_600_ = 0.4–0.5) harboring the prime editing plasmid. The infection was allowed to proceed for 10 minutes. After infection, excess bacterial suspension was removed by gently blotting the leaf surface with sterile filter paper. The explants were then transferred back to 1/2 MS solid medium and incubated for 24 hours at 25 °C in the dark to allow for co-cultivation.

Following co-culture, the leaves were washed three times with sterile water to remove residual bacteria and again blotted dry with sterile filter paper. They were then transferred to induction medium supplemented with 0.5 mg/L indole-3-butyric acid (IBA) and 400 mg/L cephalosporin to suppress bacterial overgrowth. The induction medium was refreshed every 14 days. Once hairy roots reached approximately 2 cm in length, they were excised and transferred to 6,7-V liquid medium containing 0.5 mg/L IBA and 400 mg/L cephalosporin. The liquid culture was maintained by replacing the medium every 14 days. Hairy roots were harvested after 60 days for subsequent metabolite analysis.

For the generation of transgenic plants, a similar transformation protocol was followed using *A. tumefaciens* strain EHA105 as the delivery vector. The induction medium was modified to MS solid medium supplemented with 0.1 mg/L naphthaleneacetic acid (NAA), 0.5 mg/L 6-benzylaminopurine (6-BA), and 400 mg/L cephalosporin. Additionally, 10 mg/L hygromycin was included as a selective agent for transgenic tissues. The culture environment for shoot induction was maintained at 25 °C under a 16-hour light/8-hour dark photoperiod.

**Mutagenesis analysis**

Genome DNA was extracted from hairy roots or transgenic T_0_ lines by CTAB(Murray and Thompson, 1980), and amplified from *Cas9* and *RolB* gene fragments in the treated samples by PCR, retaining positive plants with bright electrophoretic bands for subsequent mutation efficiency assessment. Further, the target fragment was PCR amplified and the PCR product was subjected to Sanger sequencing to assess the probability of gene editing events. The editing outcomes were identified by direct PCR product sanger sequencing provided by Sangon Biotech (Shanghai, China). Other mutants generated in this study and related to **Table S1** were exhibited in **Figure S6**.

**High performance liquid chromatography (HPLC) for metabolite detection**

Fresh root weight of mutant lines generated in this study was measured **(Figure S7)**. Root or hairy roots were dried in a 50°C oven, ground into fine powder and passed through a 60-mesh screen. The detection of tanshinones and salvianolic acid contents was carried out according to the method previously reported(Yu *et al.*, 2025).

For detection of tanshinones, mix 0.5 g of powdered root material with 50 mL of methanol, incubate at 25°C in an ultrasonic bath for 30 min, centrifuge at 4000 rpm for 10 min, and collect the supernatant. The chromatographic conditions are as follows: acetonitrile as mobile phase A, 0.02% aqueous phosphoric acid as mobile phase B. The flow rate is 61% for phase A and 39% for phase B within 0-6min, 90% for phase A and 10% for phase B within 6-20min, and 61% for phase A and 39% for phase B within 20-25 min. The tanshinones content is estimated based on the calibration curve of the standard Cryptotanshinone, tanshinone I, and tanshinone IIA (>98%) at a detection wavelength of 270 nm.

For detection of salvianolic acid, mix 0.1 g of powdered root material with 10 mL of 80% methanol, incubate at 25°C in an ultrasonic bath for 30 min, centrifuge at 4000 rpm for 10 min, and collect the supernatant. Using acetonitrile-0.1% aqueous phosphoric acid solution (22:78, v/v) as the mobile phase, the flow rate was controlled at 1.2 mL/min, and the elution time was 30 min. The salvianolic acid content is estimated based on the calibration curve of the standard rosmarinic acid and salvianolic acid B (>98%) at a detection wavelength of 270 nm.

The detection scheme for anthocyanins is as previous described(Zhou et al., 2025). In briefly, collect fresh petals, weigh 0.3 g of the sample, quickly freeze it with liquid nitrogen, grind it into fine powder. Using 2 mL 1.5 mol/L hydrochloric acid (85:15, v/v) as the extraction solvent, the anthocyanins were extracted with 95% ethanol and detected by 520 nm high-performance liquid chromatography. The mobile phase consists of two parts: solvent A (a water solution containing 10% formic acid) and B (a methanol solution containing 10% formic acid). The column temperature is maintained at 30℃, and the injection volume is 10 μL. The mobile phase is 1.5 mol/L hydrochloric acid (85:15, v/v). The anthocyanin content is estimated based on the calibration curve of the standard Cyanidin, Delphinidin, and Pelargonidin-3-O-glucoside (>98%) at a detection wavelength of 365 nm.

The detection scheme for rutin content is as previous described(Liu et al., 2022). In briefly, mix 0.1 g of powdered root material with 10 mL of methanol, incubate at 60°C in an ultrasonic bath for 30 min, centrifuge at 4000 rpm for 10 min, and collect the supernatant. Then filter the solution through a 0.22-μm membrane filter. The HPLC conditions are as follows: column temperature, 30°C; mobile phase A is methanol, mobile phase B is 0.5% phosphoric acid, with a volume ratio of 40:60; flow rate set at 1 mL/min; injection volume, 10 μL. The rutin content is estimated based on the calibration curve of the standard rutin hydrate (98%) at a detection wavelength of 257 nm. All solution is run on an Agilent 1260 system equipped with an C18 column (octadecyl silane bonded silica gel as filler) for HPLC.

**Real-time quantitative PCR**

The total RNA was extracted by using the *SteadyPure* plant RNA isolation kit (Accurate Biology, China), and the first strand cDNA synthesized by using the HiScript III 1st Strand cDNA Synthesis Kit (Vazyme, China). The real-time qPCR was performed using ChamQ Universal SYBR qPCR Master Mix (Vazyme, China), and the relative expression level was calculated by the 2^-ΔΔCT^ method (Livak and Schmittgen, 2001).

**Molecular docking**

The three-dimensional (3D) structures of SmRAS and FdUFGT3 proteins were predicted using the AlphaFold3 platform ([https://alphafoldserver.com/](https://alphafoldserver.com/)). The molecular structures of ligands including danshensu (PubChem CID: 11600642), rosmarinic acid (PubChem CID: 5281792), quercetin (PubChem CID: 5280343), UDP-glucose (PubChem CID: 8629), and rutin (PubChem CID: 5280805) were retrieved from the PubChem database ([https://pubchem.ncbi.nlm.nih.gov/](https://pubchem.ncbi.nlm.nih.gov/)) in .sdf format.

Protein structure files (.pdb) and ligand files (.sdf) were imported into Molecular Operating Environment (MOE) software (version 2022) for docking analysis. Prior to docking, all structures were preprocessed by removing crystallographic water molecules and adding missing hydrogen atoms. Binding pockets were defined based on predicted or known active site residues.

Flexible molecular docking was performed using MOE’s standard docking protocol. For each protein–ligand interaction, the algorithm generated the 10 most stable conformations ranked by binding free energy. The conformation with the lowest predicted binding energy was selected for further analysis.

Structural visualization of the protein–ligand complexes, hydrogen bonding interactions, and binding pockets was performed using PyMOL software (version 3.1).

**Data analysis**

The data were analyzed using GraphPad Prism 8.0 software, and the figures were further processed using Adobe Photoshop and Adobe Illustrator software. The prime editing efficiency was calculated used the formula: prime editing efficiency= (prime editing lines/total transgenic lines) *100%. The biallelic mutation rate was calculated used the formula: biallelic mutation rate = (biallelic mutation lines/total transgenic lines) *100%. The least significant difference test (LSD) of all metabolite content was used for mean separation at significance levels of 0.05 and 0.01. Each dot represents a biological replicate, and the data are presented as mean ± SD. All data were analyzed using one-way ANOVA with Dunnett’s test.

**Data availability***. Accession code.* The MediPlant-NEPE backbone pLSD801 is available from Addgene: pLSD801 (#241428)

**Reference**

**Engler, C., Gruetzner, R., Kandzia, R., and Marillonnet, S.** (2009). Golden gate shuffling: a one-pot DNA shuffling method based on type IIs restriction enzymes. PLoS One **4**:e5553. 10.1371/journal.pone.0005553.

**Livak, J K. and Schmittgen, T D.** (2001) Analysis of relative gene expression data using real-time quantitative PCR and the 2(-Delta Delta C(T)). Method, 25, 402-408. 10.1006/meth.2001.1262.

**Liu, M., Sun, W., Ma, Z., Guo, C., Chen, J., Wu, Q., Wang, X., and Chen, H.** (2022). Integrated network analyses identify MYB4R1 neofunctionalization in the UV-B adaptation of Tartary buckwheat. Plant Commun **3**:100414. 10.1016/j.xplc.2022.100414.

**Murray, M.G., and Thompson, W.F.** (1980). Rapid isolation of high molecular weight plant DNA. Nucleic Acids Res **8**:4321-4325. 10.1093/nar/8.19.4321.

**Yu, H., Liao, J., Jiang, Y., Zhong, M., Tao, S., Chai, S., Wang, L., Lin, L., Yang, R., Deng, X., et al.** (2025). Ecotype-specific phenolic acid accumulation and root softness in Salvia miltiorrhiza are driven by environmental and genetic factors. Plant Biotechnol J **23**:2224-2241. 10.1111/pbi.70048.

**Zhong, Z., Fan, T., He, Y., Liu, S., Zheng, X., Xu, Y., Ren, J., Yuan, H., Xu, Z., and Zhang, Y.** (2024). An improved plant prime editor for efficient generation of multiple-nucleotide variations and structural variations in rice. Plant Commun:100976. 10.1016/j.xplc.2024.100976.

**Zhou, H., Deng, Q., Li, M., Cheng, H., Huang, Y., Liao, J., Mo, Y., Zhao, K., Xie, Q., Ma, Y., et al.** (2025). R2R3-MYB transcription factor CaMYB5 regulates anthocyanin biosynthesis in pepper fruits. Int J Biol Macromol **308**:142450. 10.1016/j.ijbiomac.2025.142450.

**Figure S1.**

**
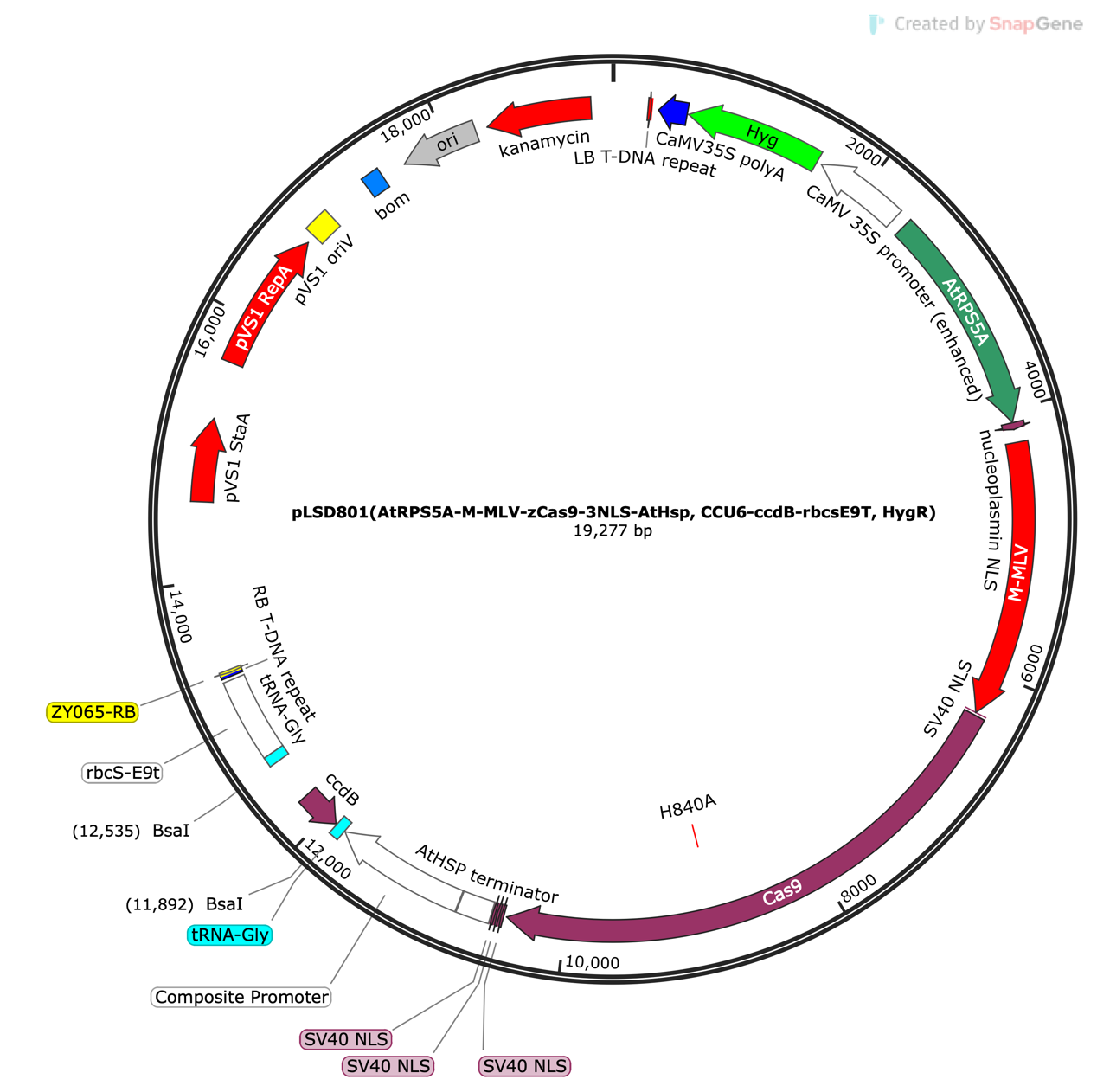
**

**Figure S1. Plasmid map in this study.**

**Figure S2.**

**
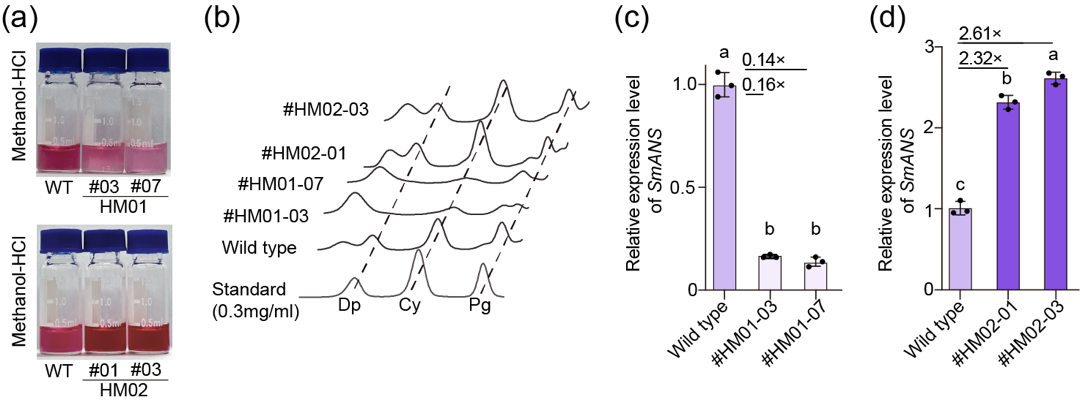
**

**Figure S2. Analysis of *SmMYB36* and *SmTCP15* mutant lines.**

**(a),** Methanol–HCl extracts of flowers from *SmMYB36* and *SmTCP15* mutant lines showing visible pigment differences. **(b),** HPLC chromatograms of flower extracts from *SmMYB36* mutants (#HM01-03 and #HM01-07) and *SmTCP15* mutants (#HM02-01 and #HM02-03). Standard concentrations for reference compounds are shown. Dp, delphinidin; Cy, cyanidin; Pg, pelargonidin. All data were showed in **Source Data** file. **(c),** Relative expression levels of *SmANS* in *SmMYB36* mutant lines (#HM01-03 and #HM01-07), determined by qRT-PCR. Each dot represents an individual technical replicate *(n=3)*. Data are shown as mean ± SD and analyzed using a two-tailed unpaired t-test. **(d),** Relative expression levels of *SmANS* in *SmTCP15* mutant lines (#HM02-01 and #HM02-03), analyzed as described in panel **c**.

**Figure S3.**

**
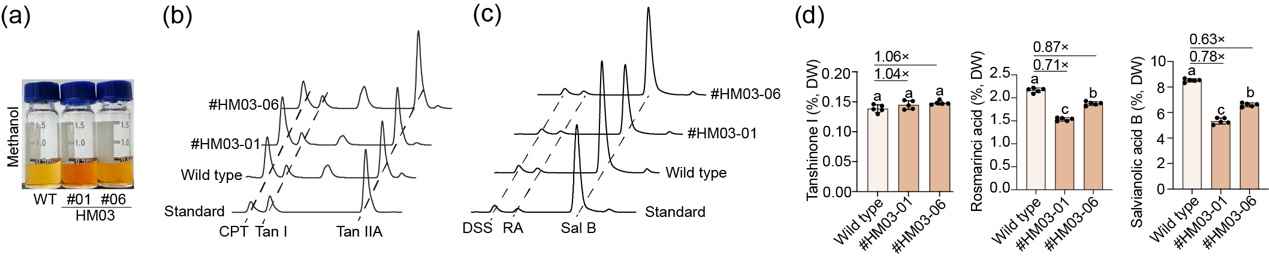
**

**Figure S3. *SmbZIP1* mutants enhances metabolite production of tanshinones in *Salvia miltiorrhiza*.**

**(a),** Methanol extracts of roots from *SmbZIP1* mutants (#HM03-01 and #HM03-06). **(b),** HPLC chromatograms of root extracts from *SmbZIP1* mutants (#HM03-01 and #HM03-06). Standard concentrations for reference compounds: cryptotanshinone (CPT), 0.05 mg/mL; tanshinone I (Tan I), 0.092 mg/mL; tanshinone IIA (Tan IIA), 0.16 mg/mL. **(c),** HPLC chromatograms of root extracts from *SmbZIP1* mutants (#HM03-01 and #HM03-06). Standard concentrations for reference compounds: danshensu (DSS), 0.05 mg/mL; rosmarinic acid (RA), 0.05 mg/mL; salvianolic acid B (Sal B), 0.05 mg/mL. All data were showed in **Source Data** file. **(d),** HPLC-based quantification of tanshinone I and phenolic acid in roots of *SmbZIP1* mutants. Each dot represents a technical replicate *(n=5)*. Data are presented as mean ± SD. Statistical significance was assessed using one-way ANOVA with Dunnett’s post hoc test.

**Figure S4.**

**
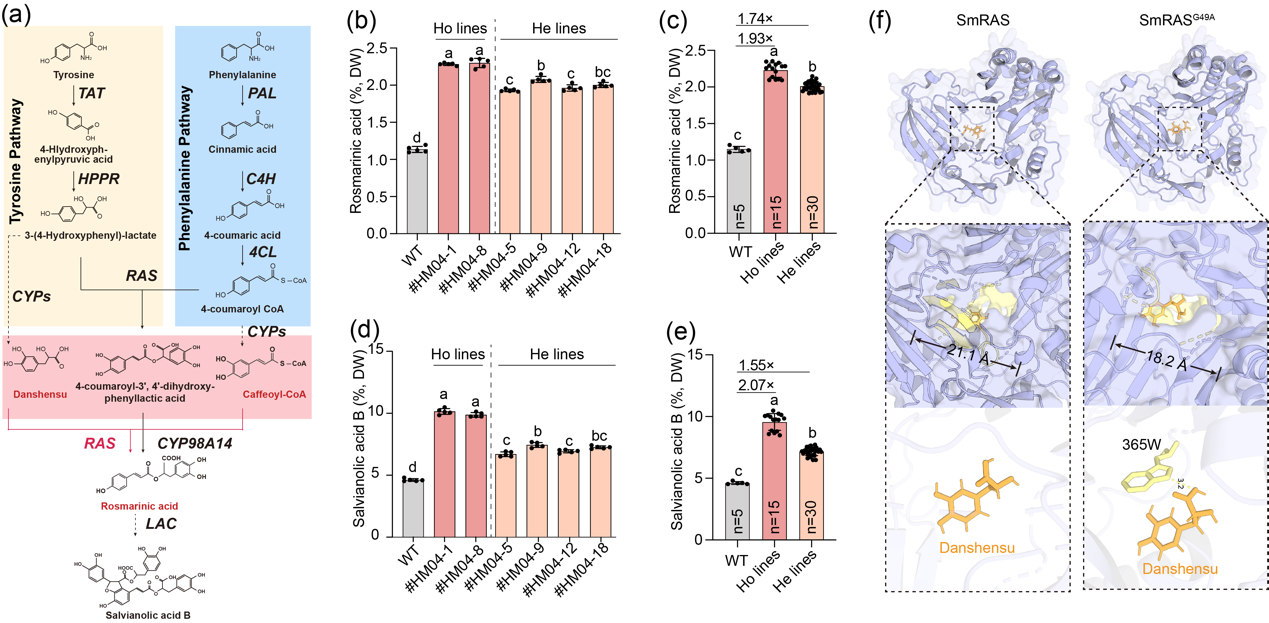
**

**Figure S4. SmRAS^G49A^ enhances metabolite production in *Salvia miltiorrhiza* hairy roots.**

**(a),** Phenolic acid biosynthetic pathways in *Salvia miltiorrhiza*. The tyrosine-derived pathway is boxed in light yellow, while the phenylalanine-derived pathway is boxed in blue. Substrates leading to the biosynthesis of rosmarinic acid are highlighted in red boxes. Key enzymes at each biosynthetic step are indicated. *RAS* and its associated metabolites are labeled in red. **(b),** HPLC quantification of rosmarinic acid in *SmRAS* mutants generated in this study. Ho, homozygous mutant lines; He, heterozygous mutant lines. **(c),** Comparison of total rosmarinic acid content between homozygous and heterozygous mutation lines. **(d),** HPLC quantification of salvianolic acid B in *SmRAS* mutants generated in this study. **(e),** Comparison of total salvianolic acid B content between homozygous and heterozygous mutation lines. Each dot represents an individual technical replicate. Data are shown as mean ± SD and analyzed using a one-way ANOVA with Dunnett’s post hoc test. **(f),** Molecular docking models of wild-type SmRAS and the SmRAS^G49A^ variant with the substrate Danshensu. Binding pocket volumes are indicated, and relevant amino acid residues involved in substrate interaction are shown.

**Figure S5.**

**
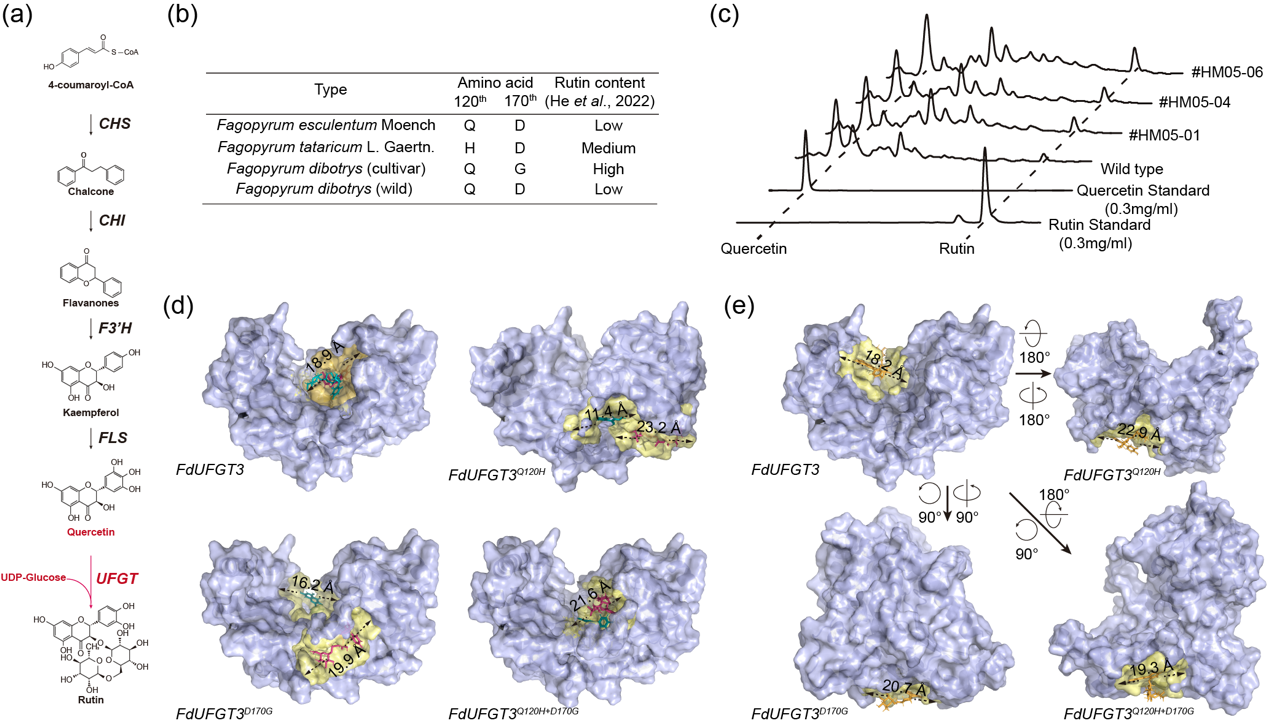
**

**Figure S5. FdUFGT3 variants enhance rutin biosynthesis in *Fagopyrum dibotrys* hairy roots.**

**(a),** Rutin biosynthetic pathway in *Fagopyrum dibotrys*. Key enzymes for each step are labeled. *UFGT* and its associated substrate are highlighted in red. **(b),** Summary of UFGT3 amino acid variations among different buckwheat cultivars with differing rutin content. Reference: He, M., He, Y., Zhang, K., Lu, X., Zhang, X., Gao, B., Fan, Y., Zhao, H., Jha, R., Huda, M.N., et al**.** (2022). Comparison of buckwheat genomes reveals the genetic basis of metabolomic divergence and ecotype differentiation. New Phytol 235:1927-1943. 10.1111/nph.18306. **(c),** HPLC chromatograms showing rutin accumulation in wild-type and *FdUFGT3* variant hairy root lines. All data were showed in **Source Data** file. **(d),** Molecular docking results of wild-type FdUFGT3 and its variants (FdUFGT3^Q120H^, FdUFGT3^D170G^, and the double mutant FdUFGT3^Q120H+D170G^) with the substrate quercetin and UDP-glucose. Quercetin is shown in blue, and UDP-glucose in red. Enzyme-substrate interactions and binding pocket volumes are visualized using PyMOL. **e,** Docking models of wild-type FdUFGT3 and the same variants described in **(d)** with the final product rutin. Rutin is displayed in orange. Protein-product conformations and active site volumes were visualized using PyMOL.

**Figure S6.**

**
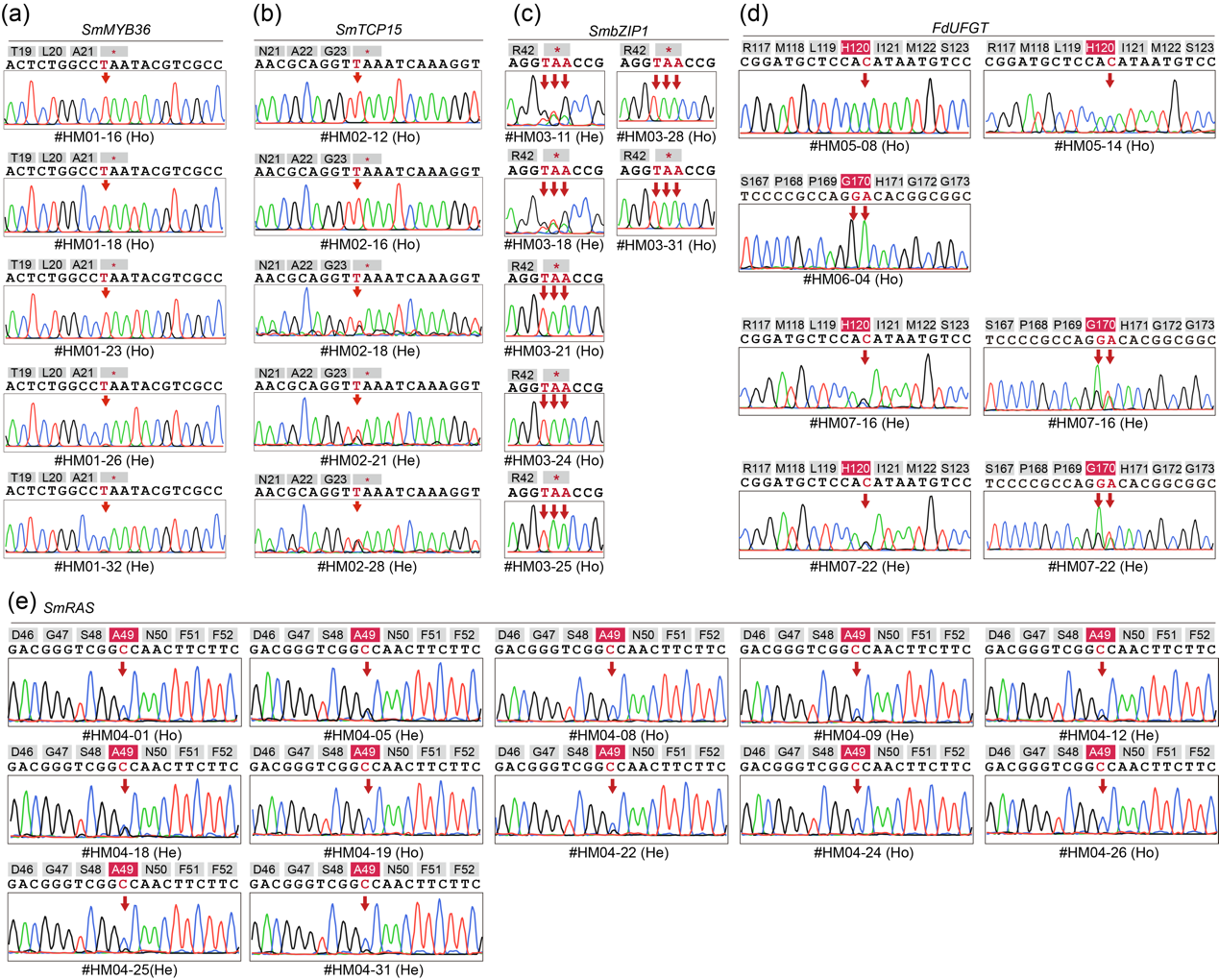
**

**Figure S6. Sanger sequencing results of additional mutant lines generated in this study.** Red arrows indicate the position of the intended nucleotide edits.

**Figure S7.**

**
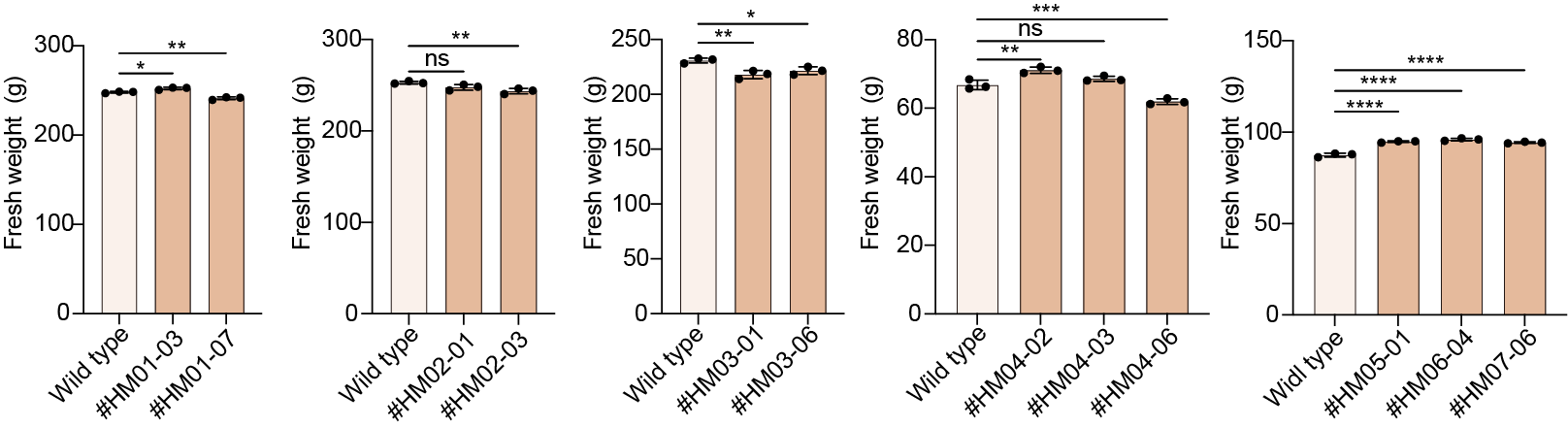
**

**Figure S7. Fresh root weight of mutant lines generated in this study.** Each dot represents a technical replicate *(n=3)*. Data are presented as mean ± SD. Statistical significance was assessed using one-way ANOVA with Dunnett’s post hoc test. ns, *P*>0.05; *, *P*<0.05; **, *P*<0.01; ***, *P*<0.001; ****, *P*<0.0001.

**Table S1. Prime editing efficiency of MediPlant-NEPE system.**

| **Vector No.** | **Target gene** | **Modification** | **No. of desired edits** | **No. of transgenic lines** | **Prime editing efficiency (%)** | **Biallelic mutation rate (%)** |
| --- | --- | --- | --- | --- | --- | --- |
| #HM01 | *SmMYB36* | C to T, Q22* | 7 | 33 | 21.21 | 15.15 |
| #HM02 | *SmTCP15* | G to T, E24* | 7 | 33 | 21.21 | 12.12 |
| #HM03 | *SmbZIP1* | GTG to TAA, V43* | 9 | 34 | 26.47 | 17.65 |
| #HM04 | *SmRAS* | G to C, G49A | 15 | 38 | 39.47 | 15.79 |
| #HM05 | *FdUFGT3* | G to C, Q120H | 3 | 25 | 12.00 | 12.00 |
| #HM06 | *FdUFGT3* | AT to GA, D170G | 2 | 25 | 8.00 | 8.00 |
| #HM07 | *FdUFGT3* | Q120H+D170G | 3 | 25 | 12.00 | 4.00 |

The amino acid change was labeled in red

**Table S2. Details of prime editing sequences in this study.**

| **Target gene** | **Modification** | **Protospacer** | **RTT** | **PBS** |
| --- | --- | --- | --- | --- |
| *SmMYB36* | C to T | TGTGGATGAAGATACGACTC | GACGTATTaGGCCA | GAGTCGTATCTT |
| *SmTCP15* | G to T | AGCAGCAACAACAACAACGC | CTTTGATTTaACCT | GCGTTGTTGTTG |
| *SmbZIP1* | GTG to TAA | AGAGTGATGATGAGATCAGA | ATCTCCGGttaCCT | TCTGATCTCATC |
| *SmRAS* | G to C | TTTCTACCGGCACGACGGGT | CGAAGAAGTTGgCCG | ACCCGTCGTGC |
| *FdUFGT3* | G to C | TCTCCACCTCGTCGATTGCC | TATgTGGAGCATCCG | GGCAATCGACGA |
| *FdUFGT3* | AT to GA | CTTACTCCCTCACCCTCTCC | GTGtcCTGGCCG | GGAGAGGGTGAG |

**Table S3. Oligos used in this study.**

| **Primer Name** | **Sequence (5'-3')** | **Purpose** |
| --- | --- | --- |
| *SmRAS*-F | ATGAAGATCGATATCACAGA | Primer for Sanger sequencing at *SmRAS* gene |
| *SmRAS*-R | CTGAAAAAGCATGAGCGGGT |  |
| *SmbZIP1*-F | ATGCAAGAGCAAGCCACGA | Primer for Sanger sequencing at *SmbZIP1* gene |
| *SmbZIP1*-R | AACCTTTTTAACCTCTTGT |  |
| *SmTCP15*-F | ATGGATCCGAAGAACTCAAA | Primer for Sanger sequencing at *SmTCP15* gene |
| *SmTCP15*-R | CTTCGGAGCCAGAGCGTGCT |  |
| *SmMYB36*-F | ATGGCGAGTGATGCATCTCT | Primer for Sanger sequencing at *SmMYB36* gene |
| *SmMYB36*-R | ACCTAAGAATCAAGTCCTCT |  |
| *FdUFGT3*-F | TGATCGCCACATTCCCGGAT | Primer for Sanger sequencing at *FdUFGT3* gene |
| *FdUFGT3*-R | CTCCTCCGGcGGAGGGGTGT |  |
| *SmMYB36*-qPCR-F | AGCAGCAGGCGCTCATATAC | Primer for qPCR of *SmMYB36* gene expression |
| *SmMYB36*-qPCR-R | ACTCTTACCGCACCTGTTGA |  |
| *SmTCP15*-qPCR-F | GTCCTCCTCGTCATCCTCTG | Primer for qPCR of *SmTCP15* gene expression |
| *SmTCP15*-qPCR-R | TGTGATAGGCCAAGCTCCAA |  |
| *SmActin*-F | GTTTGTGACAATGGAACTGGAA | Primer for qPCR of *SmActin* gene expression |
| *SmActin*-R | ACGAAGCTCGTTGTAGAAGGTA |  |
